# Perioperative Nutrition in Abdominal Surgery: Recommendations and Reality

**DOI:** 10.1155/2011/739347

**Published:** 2011-05-22

**Authors:** Yannick Cerantola, Fabian Grass, Alessandra Cristaudi, Nicolas Demartines, Markus Schäfer, Martin Hübner

**Affiliations:** Department of Visceral Surgery, University Hospital CHUV, Rue du Bugnon 46, 1011 Lausanne, Switzerland

## Abstract

*Introduction*. Preoperative malnutrition is a major risk factor for increased postoperative morbidity and mortality. Definition and diagnosis of malnutrition and its treatment is still subject for controversy. Furthermore, practical implementation of nutrition-related guidelines is unknown. *Methods*. A review of the available literature and of current guidelines on perioperative nutrition was conducted. We focused on nutritional screening and perioperative nutrition in patients undergoing digestive surgery, and we assessed translation of recent guidelines in clinical practice. *Results and Conclusions*. Malnutrition is a well-recognized risk factor for poor postoperative outcome. The prevalence of malnutrition depends largely on its definition; about 40% of patients undergoing major surgery fulfil current diagnostic criteria of being at nutritional risk. The *Nutritional Risk Score* is a pragmatic and validated tool to identify patients who should benefit from nutritional support. Adequate nutritional intervention entails reduced (infectious) complications, hospital stay, and costs. Preoperative oral supplementation of a minimum of five days is preferable; depending on the patient and the type of surgery, immune-enhancing formulas are recommended. However, surgeons' compliance with evidence-based guidelines remains poor and efforts are necessary to implement routine nutritional screening and nutritional support.

## 1. Introduction

The World Health Organization cites malnutrition as the greatest single threat to the world's public health. Indeed, the reported in-hospital prevalence of malnourished patients on admission ranges up to 50% [1–5]. Increasing evidence has been accumulated during recent years that nutritional screening and therapy are important adjuncts in modern surgical care since up to 40% of patients are at nutritional risk preoperatively [6–8]. Malnutrition before gastrointestinal (GI) surgery is caused by decreased oral food intake, preexisting chronic disease, tumour cachexia, impaired absorption due to intestinal obstruction, and previous surgical bowel resection. Moreover, low socioeconomical status, as often seen in elderly and handicapped patients, represents an additional risk factor [7, 9]. 

Malnourished patients have a significantly higher morbidity and mortality, a longer length of stay (LOS) and increased hospital costs [1, 6, 7, 10, 11]. Perioperative nutrition has been convincingly shown to improve clinical outcome in patients undergoing major GI surgery and to reduce costs [1, 12]. The mechanism of action seems to be not only an improved nutritional status by providing a higher caloric intake, but primarily a reenforced immune response; nutritional formulas containing immune-modulating agents (glutamine, arginine, n-3 fatty acids, and ribonucleic acids) are particularly beneficial modulators of the acute stress response [13, 14]. Various original studies and comprehensive guidelines have been issued recently to define preoperative screening and to standardize perioperative nutrition with regard to mode, timing, duration, and formula [15]. Furthermore, there are only scarce data assessing the practical implementation of these evidence-based recommendations. 

The aim of this study was to assess the current evidence for nutritional screening as well as perioperative nutrition in major abdominal surgery and its implementation in daily clinical practice. Furthermore, a pragmatic algorithm for evidence-based perioperative nutrition is provided.

## 2. Methods

### 2.1. Data Sources and Search Strategies

Relevant articles were identified searching Medline (through PubMed) by use of the appropriate MeSH terms for the following search items: malnutrition, nutritional screening, nutritional risk, perioperative (pre-, postoperative) nutrition (oral, enteral, and parenteral), immunonutrition, practical implementation of nutritional screening, and supporting *AND* major GI surgery *AND* clinical outcome (complications, mortality, andhospital stay). Hand-searched electronic links and references of selected articles were cross-checked. The search was limited to studies published between January 1980 and June 2010 as no frequently cited milestone articles on perioperative nutrition have been published before. Only articles published in English were considered eligible [16].

### 2.2. Study Selection

We privileged systematic reviews and meta-analyses from high-impact peer-reviewed journals and recent evidence-based guidelines. Further, important original studies adding complementary information were included. Selected studies had to treat the clinical impact of either (i) *malnutrition*, or (ii) *nutritional screening* (iii), or *perioperative nutrition*, or (iv) the practical implementation of nutritional screening and support in *digestive surgery*. For each of these areas, two authors independently performed the literature search; studies of interest were identified by screening of title, abstract, or medical subject headings. Final decision on inclusion was made based on the full text articles by the entire research team.

## 3. Results

The electronic search of the literature identified more than a thousand possible hits. These were carefully screened, and irrelevant studies were excluded by title, abstract, or full text analysis. Covering a large thematic array, many eligible studies fulfilled the inclusion criteria. Therefore, a further selection was necessary based on quality and importance for our aims. Finally, we included 68 publications, of those, 14 reviews/guidelines and 36 randomized controlled trials have been identified as major contributions to the field of perioperative nutrition.

### 3.1. Definition and Diagnosis of Malnutrition

Since there are no standardized and widely accepted definitions, precise diagnosis of malnutrition remains difficult. This major methodological shortcoming contributes to the heterogeneity of studies and also impairs proper assessment of malnutrition in daily clinical practice. Diagnostic criteria range from simple patient's data, such as amount of food intake, weight loss [18], or body mass index, to biochemical markers (albumin [19], prealbumin [20]) or various physiologic assessments. In order to develop simple, reliable, and reproducible screening tools, these parameter are often combined in scores (i.e., nutritional risk index (NRI) [21]) to grade the severity of malnutrition. Questionnaires such as the subjective global assessment (SGA) [22] are also described. Biometrical analyses, such as the phase angle (PA) [23] which quantifies body lean mass and fat by electrical impedance, are less frequently used (Table 2). 

The most valuable tool for nutritional screening for surgical patients is currently the Nutritional Risk Score (NRS) that is officially recommended by the European Society of Parenteral and Enteral Nutrition (ESPEN) [17]. It is based on the amount of malnutrition, as defined by weight loss, food intake, and BMI, as well as on the severity of disease (Table 1). Its predictive value was validated by applying it to a retrospectively 128 RCTs on nutritional support [17] and prospectively in a cohort including 5051 hospitalized patients in 12 European Countries and 26 different surgical centers [8]. The NRS used retrospectively was able to distinguish between trials with a positive effect of perioperative nutritional support versus those with no effect. When applied prospectively, it showed that “at-risk” patients had more complications, higher mortality, and longer lengths of stay than “not-at-risk” patients, and these variables were significantly related to components of NRS-2002, also when adjusted for confounders. The prevalence reported of patients at risk evaluated by NRS varies in literature from 14 to 32.6% [7, 8, 24].

Since the objective in diagnosing malnutrition is to treat it as early as possible in order to improve patient's outcome, screening tools have to be correlated to postoperative outcome. In the comparison of Antoun et al., who evaluated several screening system, only serum albumin <30 g/L showed a significant association to postoperative morbidity after multivariate analysis [19]. Schiesser et al. undertook a comparison between the NRS, NRI, and PA. These methods were well correlated for diagnosis of malnutrition. Moreover, they had a predictive value for postoperative complications. The strongest correlation for the diagnosis of malnutrition was found between NRS and NRI, but only NRS was able to reliably predict postoperative morbidity after multiple regression analysis [23].

### 3.2. Treatment of Malnutrition

Perioperative malnutrition is considered as a modifiable and treatable cause of postoperative morbidity [25, 26]. While nutritional support has shown to reduce infections, complications, LOS, and costs [27–29], many questions remain concerning patient selection, timing, route of administration, and type of nutritional support remains to be elucidated.

#### 3.2.1. Patient Selection

Patients are considered to be at severe nutritional risk if the NRS is ≥3 or if at least one of the following criteria is fulfilled: weight loss of 10–15% within 6 months, BMI < 18.5 kg/m^2^, Subjective Global Assessment Grade C or Serum albumin <30 g/L [26, 30]. For these patients, major surgery should be postponed until nutritional status has been corrected [26]. 

Most patients with GI cancer have severe malnutrition preoperatively and their immunological function is suppressed. Moreover, prolonged postoperative fasting and insufficient oral food intake may worsen preexisting malnutrition. Hence, there is an increased risk of postoperative complication, and all patients should therefore benefit from perioperative nutrition prior to major oncological surgery [29]. 

When the NRS is used, patients, with a score of 3 or more are prone to develop postoperative complications and should benefit from nutritional support [8, 23]. Since age directly influences the NRS [15], elderly patients (>70 years) must be considered as at particular risk [8]. Nutritional profile of these patients is a good prognostic factor and efforts should be made to maintain an optimal nutritional status [31].

It has been shown that even in wellnourished patients, peripoperative nutritional support positively influences postoperative outcome [25]. Enhanced recovery programs have developed for such patients, with a particular focus to minimizing preoperative fasting period and maximizing carbohydrate loading [32].

#### 3.2.2. Timing of Nutrition

The role of preoperative nutritional support is to improve undernutrition before surgery, while postoperative nutrition aims at maintaining nutritional status in the catabolic period after surgery. The timing of nutritional support is widely debated. While conventional enteral nutritional support is recommended for 10–14 days prior to major surgery in patients with severe nutritional risk to improve the nutritional state, immunonutrition (IN) is administered for 5–7 days prior to surgery to all cancer patients in order to improve immune function [26]. 

Although preoperative fasting has long been considered as a dogma, Brady et al. showed that a 2-hour fasting for clear fluids does not increase complications [33]. Nowadays, a preoperative fasting of 2 hours for fluids and 6 hours for solid food is considered as best practice and recommended by the ERAS (Enhanced Recovery After Surgery) group [32].

Postoperatively, normal oral food intake or nutrition through feeding tube should start within the first 24 hours. A recent meta-analysis evaluated early commencement of postoperative enteral nutrition (within 24 h) versus traditional management in patients undergoing gastrointestinal surgery. It was in favour of early enteral feeding following gastrointestinal surgery to reduce morbidity and mortality rates [34, 35]. The beneficial effect of early oral feeding was also shown by El Nakeeb et al. [36]. There is strong evidence that oral nutritional supplements (200 mL twice daily) given from the day of surgery until normal food intake is achieved are beneficial.

While perioperative nutritional support is recommended, some studies suggest that nutrition limited to the preoperative phase might have the same beneficial effects than combined pre- and postoperative nutrition. As far as IN is concerned, three RCTs have found no difference when comparing pre- and perioperative IN patients [13, 18, 25]. Another study compared IN given perioperatively with control patients receiving IN only postoperatively [37]. A significant decrease in postoperative complications is seen in the perioperative IN group compared to the postoperative IN group.

The optimal duration of nutritional support in the postoperative period remains unclear. While using postoperative oral nutritional supplements for 8 weeks in malnourished patients enhances recovery of nutritional status and quality of life [38], benefits for well-nourished patients are less evident [39]. Concerning postoperative IN, duration of therapy varied from 3 [40] to more than 10 days [18, 25, 41–45], with the most common duration being 7 days [13, 46–51].

#### 3.2.3. Route of Administration

Basically, nutritional support, with or without regular oral diet, can be administered in three ways: orally as oral nutritional supplements (ONSs), enterally through a feeding tube, or parenterally. As stated in the ESPEN 2006 guidelines, the enteral route should always be preferred bar if intestinal obstruction, severe shock or intestinal ischemia is present. Stratton and Elia showed that both oral nutritional supplements (ONSs) and feeding tube nutrition (FTN) were able to reduce postoperative complications in gastrointestinal (GI) surgical patients, when compared to routine care nutrition alone. However they had no influence on mortality [27]. When FTN was compared to parenteral nutrition in cancer patients undergoing surgery, those receiving enteral nutritional support had significantly less infectious complications.

Lassen et al. studied the postoperative outcome of patients undergoing major upper GI surgery. Those allowed to eat at will had less complications and shorter hospital stay than patients fed through a needle-catheter jejunostomy [52].

#### 3.2.4. Type of Supplementation

A whole variety of nutritional supplementation was identified through the electronic database search.

There is strong evidence that clear carbohydrate-rich beverage administration before midnight and 2 to 3 hours before colonic surgery ameliorates pre- and postoperative patient's status, accelerates, recovery and shortens hospital stay [32].

Immunonutrition, which contains a combination of glutamine, arginine, n-3 fatty acids, and RNA, has been evaluated in numerous studies [13, 25, 29, 41, 43, 46, 47, 50, 51, 53, 54]. A recent meta-analysis assessed the impact of IN on postoperative complications, in particular infectious complications, length of hospital, stay and mortality in patients undergoing major GI surgery. Twenty-one RCTs enrolling a total of 2730 patients were included in the meta-analysis. IN significantly reduced overall complications when used preoperatively, perioperative, or postoperatively. Patients receiving IN had less infection. The mean difference in LOS favoured IN (−2.12 (95% CI −2.97, −1.26) days). However, perioperative IN had no influence on mortality (submitted data). In all of the 9 RCTs evaluating preoperative IN, duration of supplementation was within the 5–7 days recommended range [13, 18, 25, 29, 40, 46, 55–57].

When each component of IN was studied separately, disparity was observed in the results. 

Jiang et al. compared cancer patients receiving omega 3 supplementation postoperatively for 7 days to patients receiving an isocaloric isonitrogenous diet. They found a lower incidence of infectious complications and a shorter length of stay in the treatment group. However, no significant difference could be demonstrated as far as costs are concerned [58]. A meta-analysis showed a decrease in infection rate, but no advantage in LOS or mortality [59].

While Sun et al. demonstrated that branched chain amino acid enriched total parenteral nutrition reduced postoperative complications in malnourished patients with gastrointestinal cancer undergoing major surgery [60], Gianotti et al. failed to improve the clinical outcome of patients receiving perioperative amino acids [61]. In another RCT, parenteral glutamine supplementation in the preoperative period failed to decrease infection rate, wound complication, days in the intensive care unit, and mortality [62].

### 3.3. Implementation of Current Guidelines in Clinical Practice

Implementation of nutritional support strategies into daily clinical practice encounters many difficulties and considerable efforts are needed to be successful. It has been shown in several studies that malnutrition is either not recognized or not viewed as clinically significant and that appropriate interventions are not considered necessary [3, 11].

A recent one-day multinational cross-sectional European audit showed that instruments used to identify undernourished patients and those at risk differ widely. Often, national and validated tools are replaced with locally developed ones. Many countries do not implement the recommended screening policy, which leads to underdiagnosis and undertreatment of malnutrition [63].

Our group conducted a survey among Swiss and Austrian public hospitals in order to get information about implementation of the above-mentioned current guidelines. We inquired about nutritional screening and therapy and appraisal of current evidence of perioperative nutritional support.

Conforming to previous data, we observed that implementation of current guidelines was modest at best. Only 20% of the participating centres routinely screened their GI surgery patients for nutritional status. Great disparities existed regarding screening methods. Approximately two thirds of centres were using various combinations of clinical and laboratory parameters to assess patients' nutritional status. In our study, the NRS was only used by 14% of centres.

Nutritional treatment was part of perioperative care in about 70% of all centres, and mostly dedicated to cancer patients or patients undergoing major surgery rather than to patients previously screened for their nutritional risk.

Overall, about two thirds of all centres estimated that there is enough scientific evidence in favour of preoperative nutritional support. Reduced complication rates and decreased length of hospital stay were acknowledged as major advantages. Logistic and financial issues were mentioned as reasons against the implementation of nutritional support in daily clinical practice (submitted data).

## 4. Discussion

The present paper summarizes the current evidence on preoperative nutritional screening and perioperative nutrition in major abdominal surgery. Malnutrition is a common problem in GI surgery patients (40%) and doubtlessly one of the most important risk factors for postoperative complications. The NRS is a validated screening tool that reliably identifies patients at nutritional risk who benefit from a nutritional supplementation. Recent high-quality studies have delivered convincing evidence that perioperative nutrition is a highly effective treatment that entails reduced complications, hospital stay, and costs. Most impressive results have been obtained by preoperative administration of immunonutrition. 

The recent data permitted to issue actual evidence-based guidelines in an attempt to standardize perioperative nutrition in abdominal surgery. We outlined, however, that implementation of these recommendations is not satisfactory. 

In a recent survey (unpublished data), most responding surgeons acknowledged clearly the positive impact of perioperative nutrition on postoperative outcome. Nevertheless, cost issues for outpatient nutrition and time restraints are obviously prominent reasons against nutritional care. The formation of specialized multidisciplinary teams failed to improve nutritional care. It can be therefore assumed that the individual surgeon is the most straightforward way to increase adherence to nutritional guidelines!

**Figure 1:**
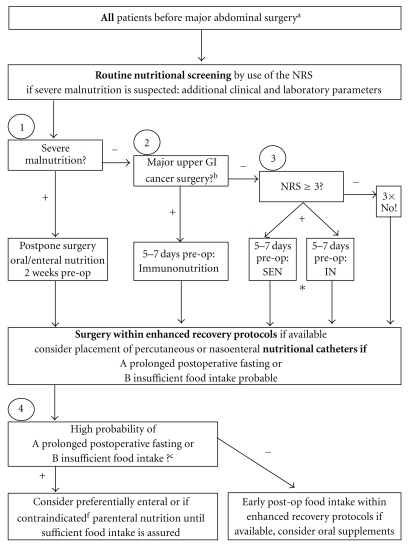
Pragmatic algorithm for preoperative nutritional screening and perioperative nutrition in digestive surgery. The algorithm resumes perioperative care in terms of nutrition in major abdominal surgery. It is largely based on recent systematic reviews and guidelines on perioperative nutrition [26, 27] and enhanced recovery [32]. ^a^Major abdominal surgery includes colorectal, gastric, liver, pancreatic, and esophageal resection for benign and malignant disease by either laparotomy or laparoscopic approach, lasting usually >2 h. ^b^Major upper GI surgery indicating preoperative IN regardless of nutritional status include oesophageal, gastric and pancreatic resection for cancer [26]. ^c^defined as anticipated perioperative starving >7 days and oral intake <60% of recommended for >10 days [26]. NRS: Nutritional Risk Score; pre-OP: pre-operative, IN: immunonutrition, SEN: standard enteral nutrition (usually whole protein formula). *currently evaluated by (http://www.clinicaltrial.gov; trial # NCT005122).

Based on the current literature and guidelines, we propose a simple and pragmatic algorithm for preoperative nutritional screening and perioperative nutritional therapy (Figure 1). All patients undergoing major surgery should be screened for malnutrition. Depending on the degree of malnutrition and the type of surgery, nutritional support should start within 14–7 days preoperatively. If insufficient postoperative food intake is anticipated, early enteral tube feeding should be started.

In conclusion, malnutrition is a well-known major risk factor for poor postoperative outcome. Preoperative nutritional screening is therefore mandatory to identify patients who need perioperative nutritional support. For most patients, a preoperative oral supplementation by whole protein formulas or immunonutrition is sufficient. The proven benefits for the patients justify the considerable efforts to foster implementation of these current guidelines in clinical practice.

##  Conflict of Interests

The authors declare that there is no conflict of interests.

## Figures and Tables

**Table 1:** Nutritional Risk Screening score (NRS 2002) [17]. The total score is obtained by adding the nutritional score to the disease score. Age > 70 years adds 1 to the total score. If age-corrected total is ≥3, the patient presents severe malnutrition, and nutritional support is recommended.

Malnutrition		Mild	Moderate	Severe
		Score 1	Score 2	Score 3
Nutritional Status	BMI (kg/m^2^)	—	18.5–20.5	<18.5
	Food Intake (%)	50–70	25–50	<25
	Weight loss <5%	3 months	2 months	1 month

Disease severity	Example	Hip fracture, cirrhosis, COPD	Major surgery^a^, Stroke	Head injury, bone marrow transplantation, ICU patients (APACHE 20)

Age	(Years)	>70		

^a^Major abdominal surgery includes colorectal, gastric, liver, pancreatic, and esophageal resection for benign and malignant disease by either laparotomy or laparoscopic approach, lasting usually >2 h.

**Table 2:** Overview on common screening tools for malnutrition and its reported prevalence depending on study and screening tool.

	Antoun et al.					Schiesser et al.		
Malnutrition	Weight loss	BMI (kg/m^2^)	SGA	Albumin (g/L)	NRI	NRS (2002)**	PA	NRI
None	**—** (29%)^1^	**18.5–25 **(50%)^1^	**A** (66%)^1^	**>35**	**>97.5 **(59%)^1^	Score **0**	**>6°** (71%)^2^	**>97.5 **(85%)^2^
Mild	**<10%** (39%)^1^	**<18.5** (8%)^1^	**B** (22%)^1^	**<35** (24%)^1^	**84–97.5 **(32%)^1^	Score **1 **(89%)^2^	**<6°** (28%)^2^	**84–97.5** (13%)^2^
Moderate						Score **2 **(8.5%)^2^		
Severe	**≥10%** (20.5%)^1^	**<16** (2%)^1^	**C** (12%)^1^	**<30** (8%)^1^	**<84** (9%)^1^	Score **3** (2.5%)^2^		**<84** (2%)^2^

BMI: body mas index (kg/m^2^); SGA: subjective global assessment (weight, food intake, symptoms, and activities); NRI: nutritional risk index (recent weight loss, serum albumin); NRS (2002): nutritional risk screening score (Table 2); PA: phase angle (reactance and resistance from bioimpedance analysis).

^1^Antoun et al. [18] (prevalence %).

^2^Schiesser et al. [23] (prevalence %).

**Nutrition status score only.

## Abbreviations

IN: Immunonutrition;
RCT: Randomized controlled trial;
NRS: Nutrirional risk score;
Gl: Gastrointestinal.
